# A So-Far Overlooked Secondary Conformation State in the Binding Mode of SARS-CoV-2 Spike Protein to Human ACE2 and Its Conversion Rate Are Crucial for Estimating Infectivity Efficacy of the Underlying Virus Variant

**DOI:** 10.1128/jvi.00685-22

**Published:** 2022-06-08

**Authors:** Marc Sevenich, Joop van den Heuvel, Ian Gering, Jeannine Mohrlüder, Dieter Willbold

**Affiliations:** a Institute of Biological Information Processing (IBI-7), Forschungszentrum Jülich, Jülich, Germany; b Helmholtz Centre for Infection Research, Braunschweig, Germany; c Institut für Physikalische Biologie, Heinrich-Heine-Universität Düsseldorf, Düsseldorf, Germany; d Priavoid GmbH, Jülich, Germany; e JuStruct, Forschungszentrum Jülich, Jülich, Germany; Loyola University Chicago

**Keywords:** SARS-CoV-2, human angiotensin converting enzyme 2, protein kinetics, spike protein, surface plasmon resonance, variants of concern

## Abstract

Since its outbreak in 2019, severe acute respiratory syndrome coronavirus 2 (SARS-CoV-2) has spread with high transmission efficiency across the world, putting health care as well as economic systems under pressure. During the course of the pandemic, the originally identified SARS-CoV-2 variant has been multiple times replaced by various mutant versions, which showed enhanced fitness due to increased infection and transmission rates. In order to find an explanation for why SARS-CoV-2 and its emerging mutated versions showed enhanced transmission efficiency compared with SARS-CoV (2002), an enhanced binding affinity of the spike protein to human angiotensin converting enzyme 2 (hACE2) has been proposed by crystal structure analysis and was identified in cell culture models. Kinetic analysis of the interaction of various spike protein constructs with hACE2 was considered to be best described by a Langmuir-based 1:1 stoichiometric interaction. However, we demonstrate in this report that the SARS-CoV-2 spike protein interaction with hACE2 is best described by a two-step interaction, which is defined by an initial binding event followed by a slower secondary rate transition that enhances the stability of the complex by a factor of ~190 (primary versus secondary state) with an overall equilibrium dissociation constant (*K_D_*) of 0.20 nM. In addition, we show that the secondary rate transition is not only present in SARS-CoV-2 wild type (“wt”; Wuhan strain) but also found in the B.1.1.7 variant, where its transition rate is 5-fold increased.

**IMPORTANCE** The current SARS-CoV-2 pandemic is characterized by the high infectivity of SARS-CoV-2 and its derived variants of concern (VOCs). It has been widely assumed that the reason for its increased cell entry compared with SARS-CoV (2002) is due to alterations in the viral spike protein, where single amino acid residue substitutions can increase affinity for hACE2. So far, the interaction of a single unit of the CoV-2 spike protein has been described using the 1:1 Langmuir interaction kinetic. However, we demonstrate here that there is a secondary state binding step that may be essential for novel VOCs in order to further increase their infectivity. These findings are important for quantitatively understanding the infection process of SARS-CoV-2 and characterization of emerging SARS-CoV-2 variants of spike proteins. Thus, they provide a tool for predicting the potential infectivity of the respective viral variants based on secondary rate transition and secondary complex stability.

## INTRODUCTION

Severe acute respiratory syndrome coronavirus 2 (SARS-CoV-2) is a beta class coronavirus that was first discovered and characterized in Wuhan, China, at the end of 2019 (1, 2). Since then, it has challenged health care systems due to its rapid spread and COVID-19 transmission throughout the world. Far more than the past coronavirus pandemic, caused by SARS-CoV in 2002, the ongoing pandemic has claimed over 6.2 million lives with over 504 million total cases so far (3–5).

The SARS-CoV-2 coronavirus replication depends on a multistep process starting with the interaction of the viral trimeric spike (S) protein and human angiotensin converting enzyme 2 (hACE2) that mediates the uptake of the viral RNA into the host-cell cytoplasm. Each monomer of the S protein consists of the two functional subunits S1 and S2. The S1 subunit comprises the receptor binding domain (RBD) that interacts via a defined motif sequence (RBM) with an N-terminally located helical structure of the hACE2. The S2 subunit, however, plays a crucial role in the membrane fusion process. While the S1-hACE2 interaction allows viral attachment to the host cell surface, the S2’-site is cleaved by the human endoprotease TMPRSS2. This leads to irreversible conformational changes of the S protein that result in cell membrane fusion and viral uptake by the host cell (6). Although the viral S proteins of SARS-CoV (2002) and SARS-CoV-2 (wt; Wuhan strain) share ~76% amino acid sequence identity, SARS-CoV-2 shows an enhanced cell infectivity and human-to-human transmission efficiency compared with SARS-CoV (7, 8). Since its first appearance in 2019, the virus has undergone numerous mutational events, resulting in variants with enhanced fitness concerning their transmissibility (9, 10). A large proportion of these mutations cluster in the spike protein, where one-third of the sequence has been associated with diverse alterations (11). To date, the most widespread variants B.1.1.7 (α-variant), B.1.617.2 (δ-variant), P.1 (γ-variant), and B.1.1.529 (o-variant) have widely replaced the originally identified SARS-CoV-2 virus due to their enhanced fitness (10, 12–17).

In order to understand why certain variants increase infectivity, the process of virus contact with the cell surface and cell uptake has been drawn into the center of attention. Although a more efficient fusion process will also impact the infectiousness of the virus (18), the interaction of the spike protein with hACE2 will provide the initial contact and therefore limit the time frame for subsequent processes. To date, the kinetics of the SARS-CoV spike-hACE2 interaction have been defined widely as a one-step binding process with a monoexponential decay using a Langmuir-based 1:1 fitting model for surface plasmon resonance or biolayer interferometry experiments (6, 19–23). However, this model fails to describe the complexity of the interaction, which becomes very apparent when looking at the heterogeneity of the complex decay.

Here, we report that the interaction of the isolated monomeric SARS-CoV-2 ectodomain with hACE2 has a two-state binding mode. The additional secondary conformational transition increases the overall stability of the prefusional state dramatically and therefore potentially enlarges the time frame for the initiation of membrane fusion and viral cell entry. In addition, we characterized the secondary interaction state in the trimeric SARS-CoV-2 B.1.1.7 (α-variant) spike protein, where the conversion rate to the secondary conformational state is increased dramatically compared with the SARS-CoV-2 wild type. This observation gives insights into how the infectivity among SARS-CoV-2 mutants is modified and represents a precise and fast analysis method to predict the infectivity of novel SARS-CoV-2 variants with mutated spike protein sequences.

## RESULTS AND DISCUSSION

### The Langmuir 1:1 binding model is not sufficient to fit experimental data for spike protein binding to hACE2.

**FIG 1:**
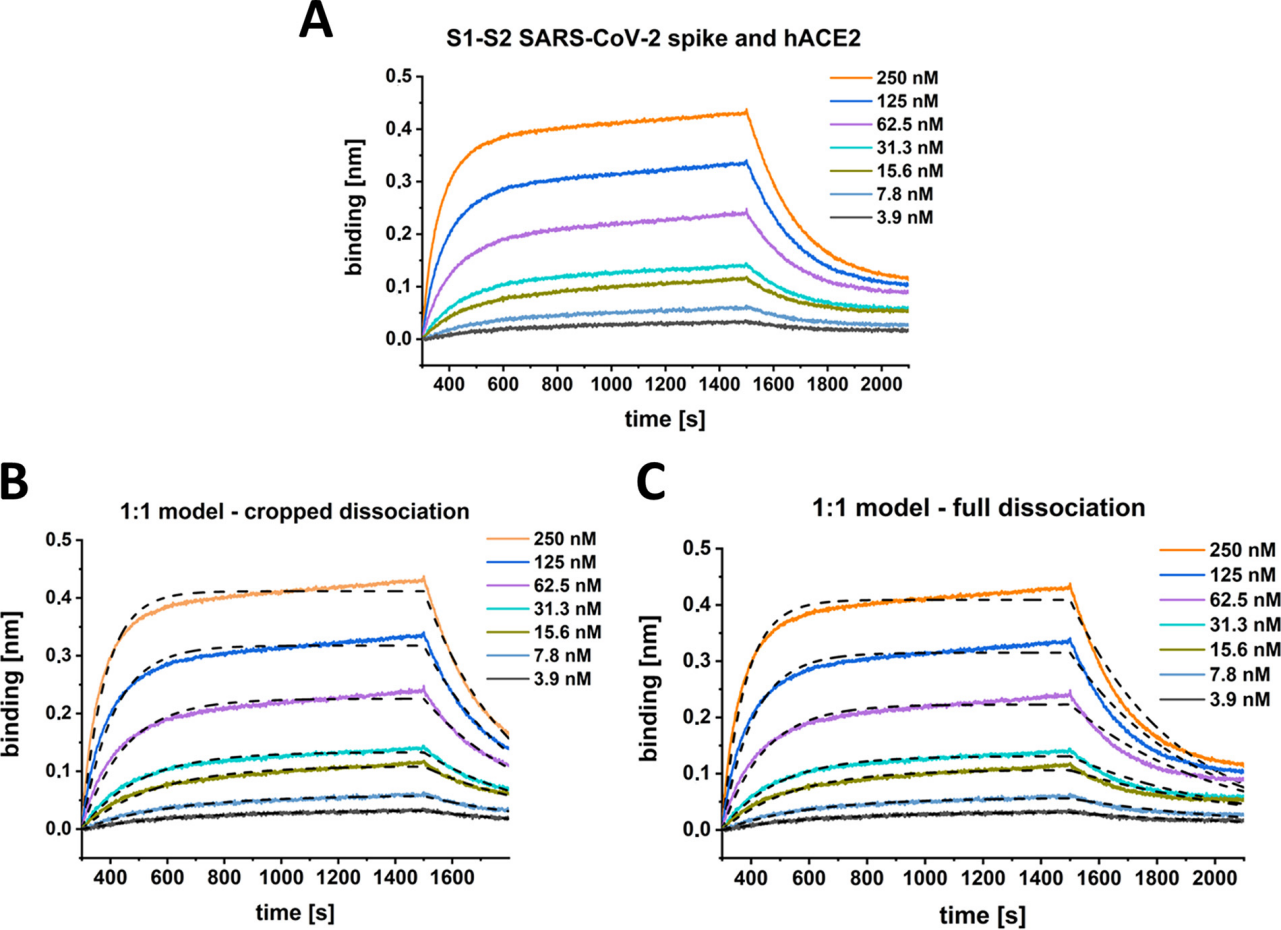
BLI kinetic experiment with SARS-CoV-2 S1-S2 and hACE2. The sensogram (A) was globally fitted with a 1:1 interaction model (B and C, black dashed lines) either with a cropped dissociation time of 200 s (B) or the full dissociation time of 600 s (C). (B) *K_D_*, 22 nM; *k_a_*, 1.1 E + 5 ± 7.5 E + 4 [1/Ms]; *k_d_*, 2.4 E-3 ± 4.7 E-3 [1/s]. R^2^, 0.98. (C) *K_D_*, 11 nM; *k_a_*, 1.7E + 5 ± 1.5 E + 5 [1/Ms], *k_d_*, 1.9 E-3 ± 4.7 E-3 [1/s]. R^2^, 0.96.

For characterization of the S1-S2 spike protein interaction with the hACE2 receptor, two different kinetic methods were applied. First, for biolayer interferometry, hACE2 was immobilized via a C-terminal biotin on a streptavidin-coated sensor surface. Incubation with a serial dilution of the S1-S2 spike protein yielded the sensograms shown in Fig. 1A. To obtain apparently good-looking fits based on a Langmuir 1:1 model, the dissociation time needed to be shortened significantly (Fig. 1B). Such incomplete fitting allowed the determination of an equilibrium dissociation constant (*K_D_*) and kinetic rates similar to those that have been published previously (6, 19, 22, 23). The model fails, however, to describe the dissociation phase of the interaction, which cannot be fitted satisfactorily by a monoexponential decay (Fig. 1 C).

**FIG 2:**
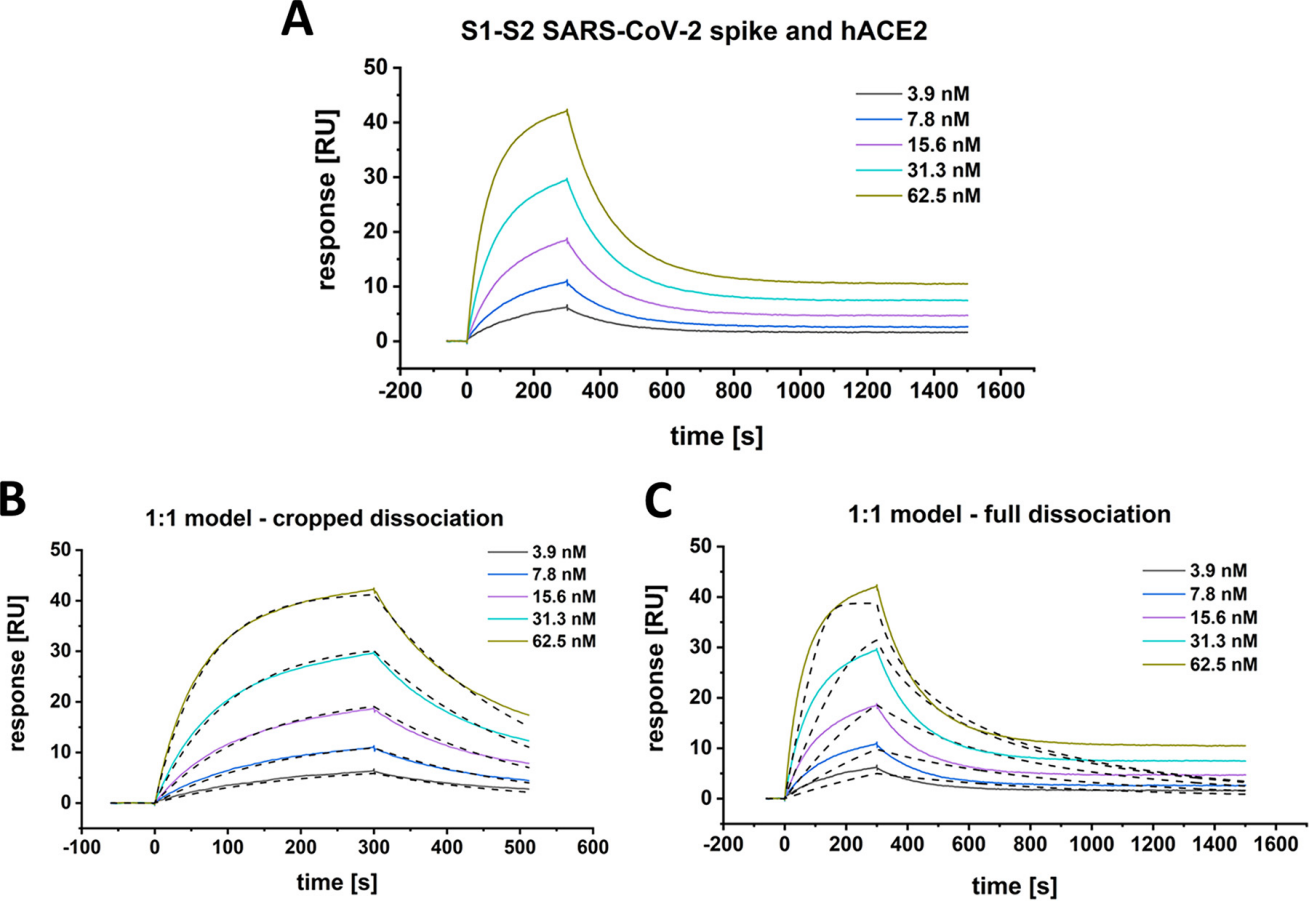
SPR-multicycle kinetic experiment of SARS-CoV-2 S1-S2 and hACE2. The sensogram (A) was globally fitted with a Langmuir 1:1 interaction model (B and C, black dashed lines) either with a cropped dissociation time of 200 s (B) or the full (C) dissociation time of 1,200 s. (B) *K_D_*, 28.5 nM; *k_a_*, 1.7 E + 5 ± 5.4 E + 2 [1/Ms]; *k_d_*, 4.7 E-3 ± 1.0 E-5 [1/s]. Chi^2^, 0.28 [RU^2^]. (C) *K_D_*, 12.2 nM; *k_a_*, 5.4 E + 7 ± 2.2 E + 6 [1/Ms]; *k_d_*, 0.7 ± 0.03 [1/s]. Chi^2^, 6.6 [RU^2^].

This conclusion is confirmed by surface plasmon resonance (SPR) experiments. hACE2 was coupled via an IgG1 fc-tag on a protein A/G derivatized surface, and various concentrations of the S1-S2 spike protein were applied as analytes (Fig. 2A). Apparently satisfying fits based on a Langmuir 1:1 model are obtained only when the dissociation time is only a few hundred seconds (Fig. 2B). The inclusion of longer dissociation times into the analysis, however, clearly shows that the interaction of the viral S1-S2 spike protein and the hACE2 is not of a 1:1 Langmuir binding model (Fig. 2C).

### S1-S2 spike protein hACE2 interaction induces a time-dependent secondary state.

**FIG 3:**
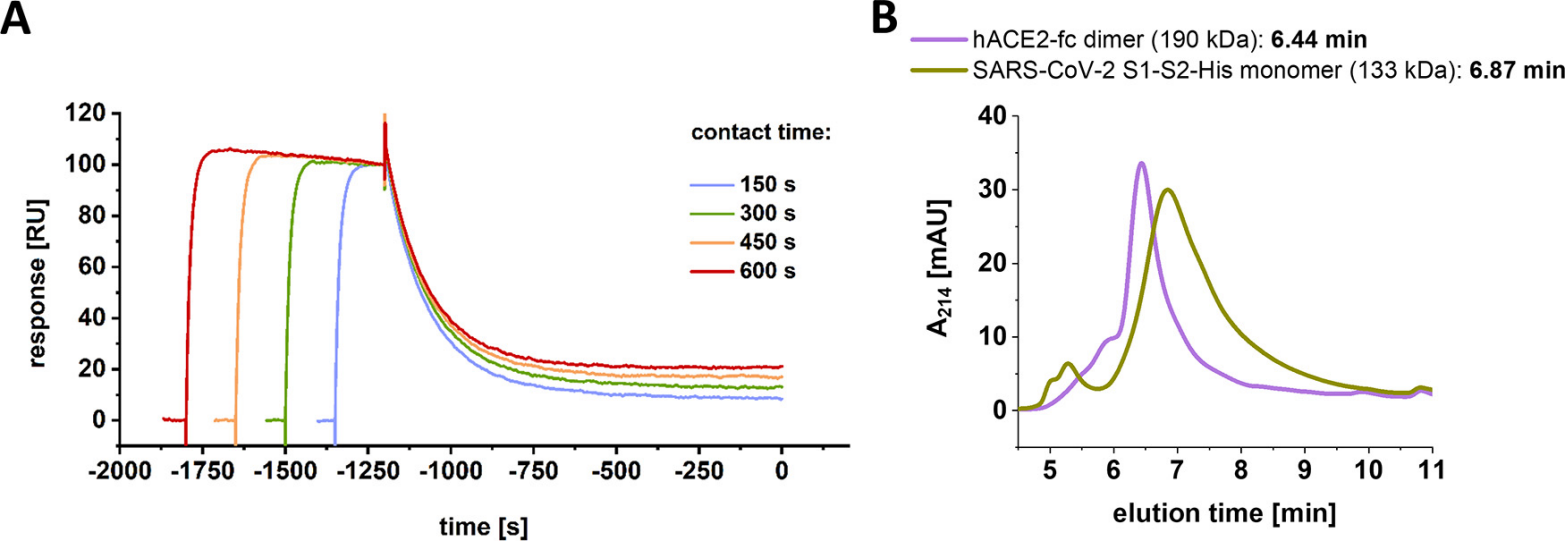
SPR experiment testing for the presence of a two-state reaction. (A) A total of 500 nM SARS-CoV-2 S1-S2 spike protein was injected over a constant immobilization level of 25 RU hACE2. Injection times were gradually increased (150 to 600 s). Dissociation start point was aligned on the time scale. (B) SEC-HPLC using 5-μg/mL injections of SARS-CoV-2 S1-S2 monomer and hACE2 dimer. Column calibration was performed using protein standards of thyroglobulin (660 kDa, 5.78 min), γ-globulin (150 kDa, 7.52 min), ovalbumin (45 kDa, 8.52 min), and aprotinin (6.5 kDa, 11.12 min).

Because the interaction of hACE2 and S1-S2 spike protein is not matching a 1:1 Langmuir binding model, we checked for the existence of a potentially underlying second rate processes by SPR (Fig. 3A) (24, 25) and verified the monomeric status of S1-S2 and the dimeric status of hACE2 by size exclusion chromatography–high-pressure liquid chromatography (SEC-HPLC).

For a test on the secondary state by SPR (Fig. 3A), hACE2 was coupled at a constant immobilization level of 25 resonance units (RU). The S1-S2 spike protein was injected at a concentration of 500 nM. Contact times were increased gradually by increasing the injection times ranging from 150 to 600 s. For each injection, steady state was reached within a short time interval so that a constant complex concentration can be assumed during the different contact times.

The resulting plot reveals a strong dependency of incubation time and dissociation rate (Fig. 3). Increasing injection times and thus increasing contact time correlate with decreasing dissociation rates. This finding cannot be expected for a single-step 1:1 interaction but clearly indicates the formation of a secondary complex state, whose proportion increases with contact time duration. This is typical for a two-step binding mode, in which the formation of the primary complex induces a conformational reorganization into a secondary complex conformation that strengthens the interaction and leads to a very low dissociation rate.

### Secondary complex state of the S1-S2 spike protein with hACE2 results in enhanced complex stability.

**FIG 4:**
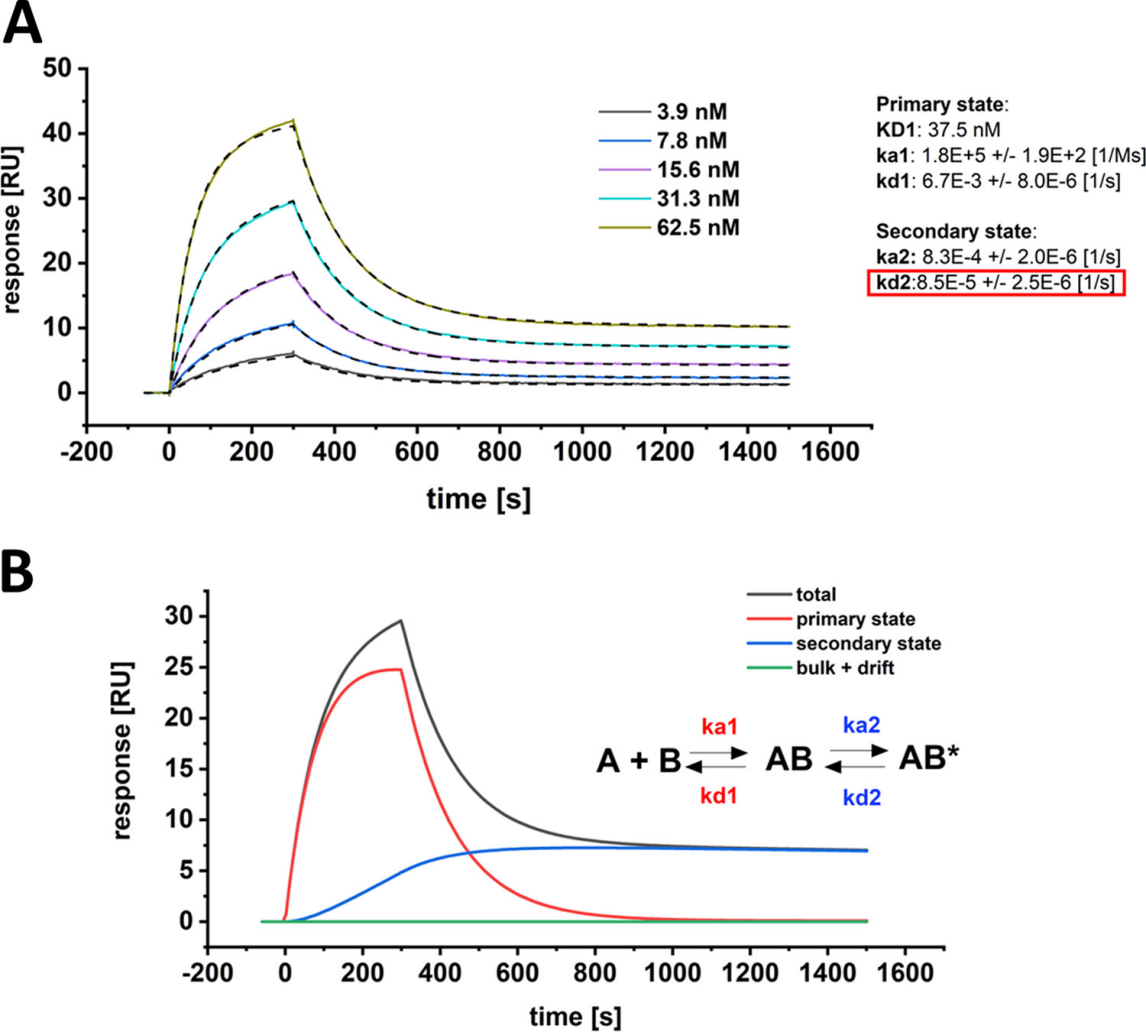
SPR multicycle kinetic experiment of SARS-CoV-2 S1-S2 and hACE2. (A) The sensogram was globally fitted with a two-state kinetic model, including the full dissociation time of 1,200 s. Kinetic parameters for the first interaction were determined with K_D1_ of 37.5 nM, k_a1_ of 1.8E + 5 ± 1.9 E + 2 [1/Ms] and k_d1_ of 6.6 E-3 ± 8.0 E-6 [1/s]. Kinetic parameters for the second interaction were determined with k_a2_ of 8.3 E-4 ± 2.0 E-6 [1/s] and k_d2_ of 8.5 E-5 ± 2.5 E-6 [1/s]. Chi^2^, 0.08 [RU^2^]. The overall K_D-total_ for both events was identified with 0.2 nM. The dissociation rate of the secondary state contributes significantly to the increase in overall affinity (red box). (B) Component analysis of the 31.3 nM S1-S2 spike protein binding curve as shown in A. The sensogram (total) is composed of the primary binding event (red), followed by a secondary transition event (blue) which results in a highly stable secondary complex (AB*).

In order to define the secondary rate kinetics of complex transition after primary binding, the interaction of the S1-S2 spike protein and hACE2 was fitted with a secondary state model. In contrast to the previously presented attempts of the Langmuir model fitting (Fig. 1 and 2), the two-step kinetic model allows the description of the interaction with high accuracy over the complete dissociation time (Fig. 4A).

The kinetic rates of the primary binding event were identified with k_a1_ of 1.8 × 10^−5^ M^−1^ s^−1^ (k_a_ = association rate or on-rate) and k_d1_ of 6.7 × 10^−3^ s^−1^ (k_d_ = dissociation rate or off-rate) resulting in a dissociation constant (*K_D_*) of 37.5 nM. This result matches with previously reported kinetic values for the RBD interaction with hACE2 (6, 19, 22, 23). As soon as the first complex is formed, a secondary event increases the complex stability (Fig. 4B). This transition is most likely a structural rearrangement that decreases the complex dynamics as proposed previously (16). The kinetic data show that this secondary process is with an on-rate of 8.3 × 10^−4^ s^−1^ rather slow compared to the primary binding event but at the same time increases the complex half-life by a factor of ~80 with an off-rate of 8.5 × 10^−5^ s^−1^. When the kinetic values of the primary and secondary events are combined to one binding constant, the full binding process yields a total affinity that is described with K_D-total_ of 0.20 nM.

### The SARS-CoV-2 RBD interaction with hACE2 follows a Langmuir interaction kinetic.

**FIG 5:**
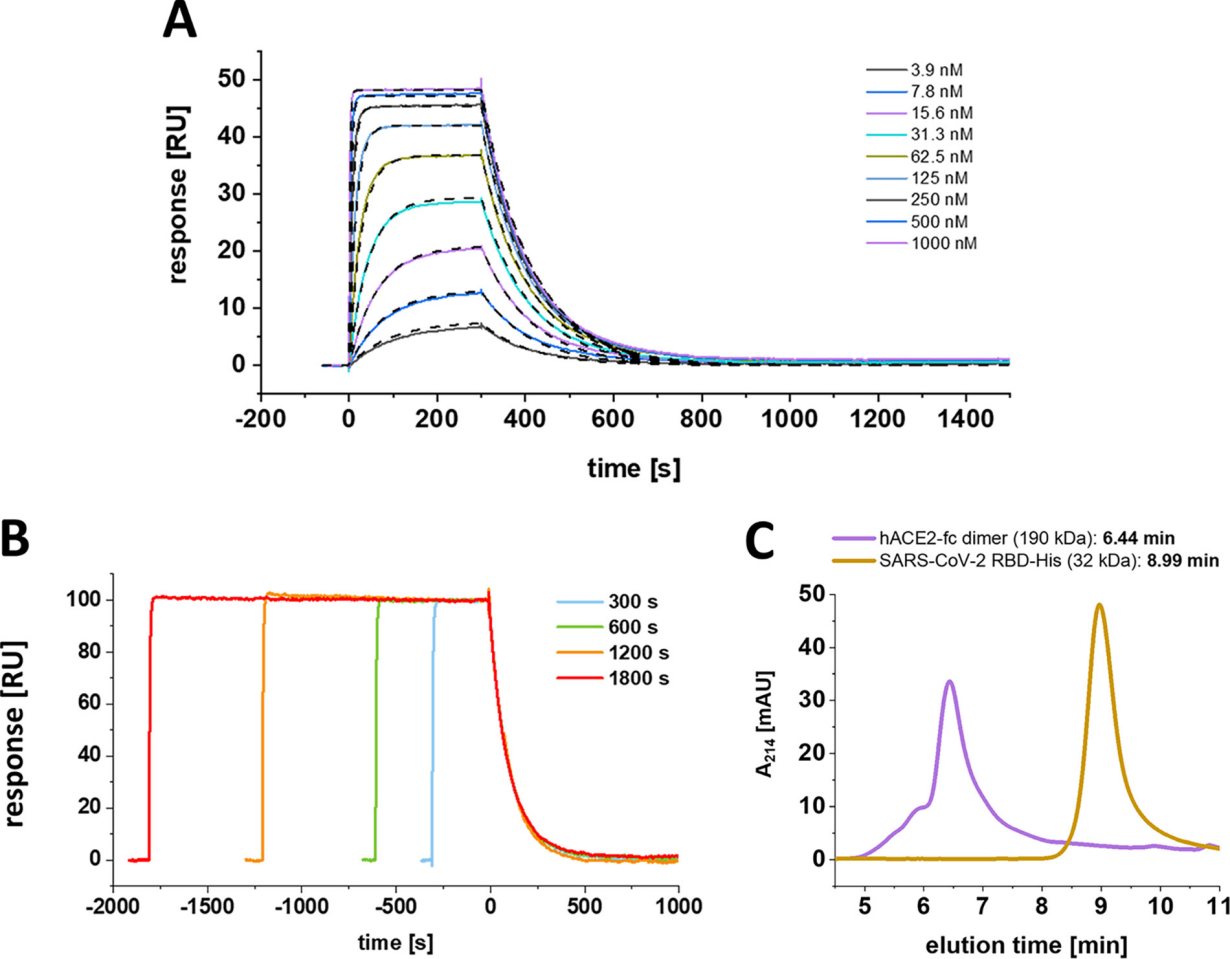
The SARS-CoV-2 RBD interaction with hACE2 follows a Langmuir-based kinetic with time-independent monoexponential decay. (A) Multicycle experiment with SARS-CoV-2 RBD. hACE2-fc was immobilized on a protein A/G sensor chip, and SARS-CoV-2 RBD was injected in a concentration range of 3.9 to 1,000 nM. The *K_D_* was globally fitted with a 1:1 Langmuir-based interaction model. The kinetic parameters were determined with a *K_D_* of 21.3 nM, *k_a_* of 4.3 E + 5 ± 2.2 E + 2 [1/Ms], and *k_d_* of 9.1 E-3 ± 4.2 E-6 [1/s]. Chi^2^, 0.05 [RU^2^] (B) Test on secondary state reaction. hACE2 was immobilized on a protein A sensor surface, and 500 nM SARS-CoV-2 was injected at a constant concentration for increasing contact intervals. Dissociation starting point was aligned on the time scale. (C) SEC-HPLC using 5-μg/mL injections of SARS-CoV-2 RBD and hACE2 dimer. Column calibration was performed using protein standards of thyroglobulin (660 kDa, 5.78 min), γ-globulin (150 kDa, 7.52 min), ovalbumin (45 kDa, 8.52 min), and aprotinin (6.5 kDa, 11.12 min).

To verify whether the secondary transition can be obtained exclusively for the S1-S2 monomeric construct or might be associated with certain proportions of the spike protein, a SARS-CoV-2 RBD construct was assayed for the existence of an underlying secondary rate kinetic (Fig. 5A and B).

After confirmation of its monomeric status (Fig. 5C), SARS-CoV-2 RBD was analyzed in a multicycle kinetic experiment on the immobilized hACE2, where the dissociation phase showed a clear monoexponential behavior with complete baseline dissociation, which is in full agreement with a Langmuir-based 1:1 interaction model (Fig. 5A). This finding appears to be in contrast with the previously identified biphasic dissociation for the S1-S2 SARS-CoV-2 construct. Additionally, the RBD did not show a contact time-dependent alteration of the dissociation phase, when increasing contact times are applied (Fig. 5B). However, the dissociation constant as well as the on- and off-rate of the RBD and the primary binding event of the monomeric S1-S2 show high similarity. This finding implies that the primary binding event of the two-state interaction is carried out by the interaction of the RBD and hACE2 alone, whereas the context of the full S1-S2 protein is essential for the formation of the secondary complex.

### The secondary state transition rate is modified among SARS-CoV, SARS-CoV-2, and B.1.1.7 trimeric spike proteins.

**FIG 6:**
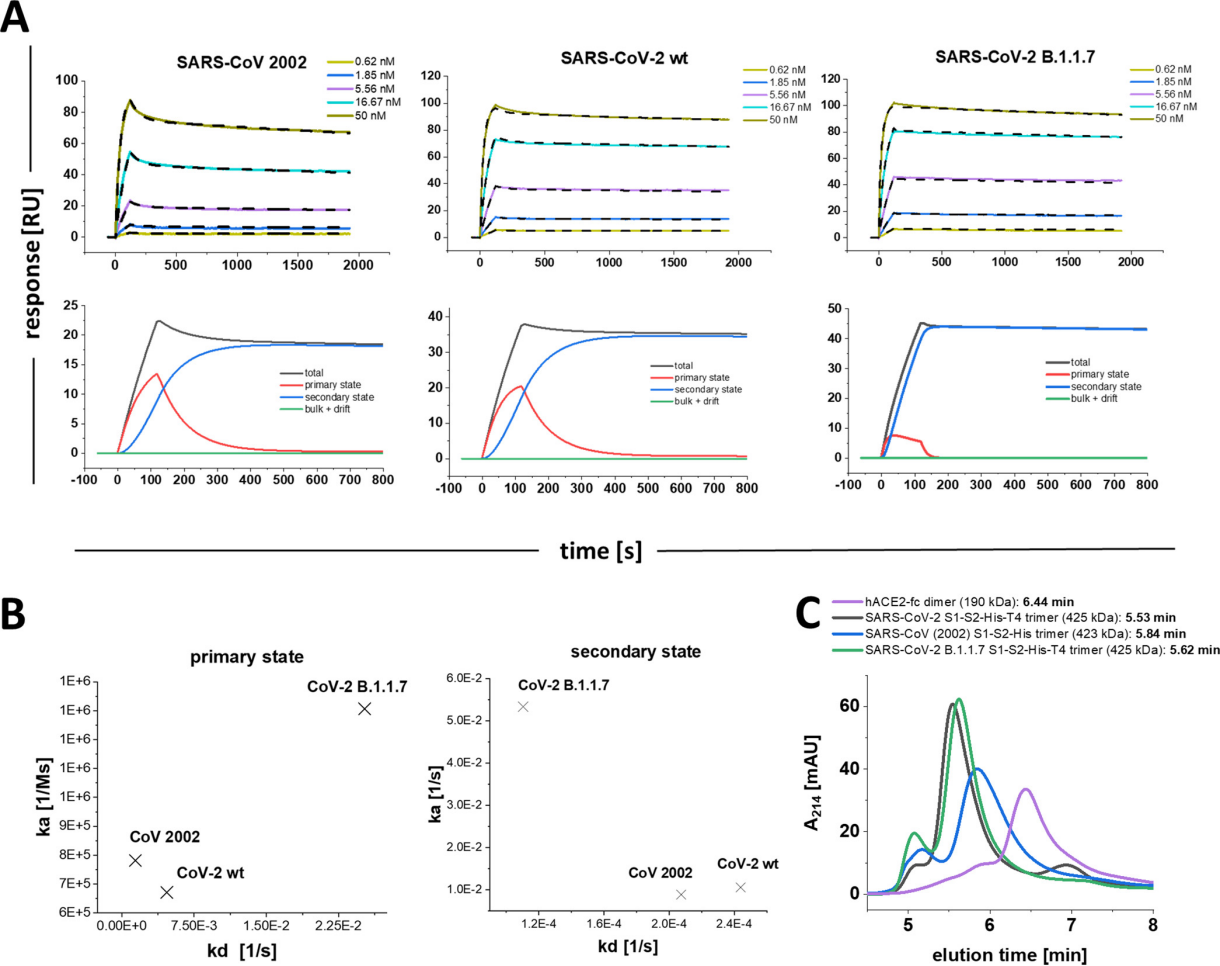
Multicycle SPR experiments with SARS-CoV (2002), SARS-CoV-2 wt, and SARS-CoV-2 B.1.1.7 trimeric spike proteins. (A) hACE2-fc was immobilized on a protein A/G sensor surface, and CoV trimer proteins were injected in concentration range of 0.62 to 50 nM. The sensograms were globally fitted with the secondary state reaction model. SARS-CoV (2002): K_D1_, 6.7 nM; k_a1_, 6.72 E + 5 ± 4.4 E + 3 [1/Ms]; k_d1_, 4.7 E-3 [1/s] ± 6.9 E-5; k_a2_, 8.8 E-3 ± 6.9 E-5 [1/Ms]; k_d2_, 2.1 E-4 ± 1.6 E-6 [1/s]; K_D-total_, 160 pM. Chi^2^, 0.6 [RU^2^]. SARS-CoV-2 wt: K_D1_, 1.8 nM; k_a1_, 7.8 E + 5 ± 1.2E + 3 [1/Ms]; k_d1_, 1.41 E-3 ± 3.75E-5 [1/s]; k_a2_, 1.1 E-2 ± 1.8 E-4 [1/Ms]; k_d2_, 2.4 E-4 ± 3.6 E-6 [1/s]; K_D-total_ 41 pM. Chi^2^: 0.8 [RU^2^]. (SARS-CoV-2 B.1.1.7) K_D1_, 193 nM, k_a1_, 1.31 E + 6 ± 1.5 E + 4 [1/Ms]; k_d1_, 2.5 E-2 ± 1.5 E-3 [1/s]; k_a2_, 5.3 E-2 ± 1.5 E-3 [1/Ms]; k_d2_, 1.1 E-4 ± 2.6 E-6 [1/s]; K_D-total_, 40 pM; Chi^2^, 0.7 [RU^2^]. For graphic representation of the distribution of primary and secondary state reaction, a component analysis was performed for each of the trimeric spike proteins using the injection concentration of 5.56 nM spike. The sensogram (total) is composed of the primary binding event (red), followed by a secondary transition event (blue) which results in a highly stable secondary complex (B) On-off chart for the kinetic values of the SARS-CoV spike trimers in the primary and secondary state. (C) SEC-HPLC using 5-μg/mL injections of SARS-CoV-2 S1-S2 trimer, SARS-CoV (2002) trimer, SARS-CoV-2 B.1.1.7 trimer, and hACE2 dimer. Column calibration was performed using protein standards of thyroglobulin (660 kDa, 5.78 min), γ-globulin (150 kDa, 7.52 min), ovalbumin (45 kDa, 8.52 min), and aprotinin (6.5 kDa, 11.12 min).

The secondary state model was essential to fully describe the interaction of the S1-S2 monomer with hACE2. Next, we analyzed the interaction of the SARS-CoV (2002), SARS-CoV-2 wt, and SARS-CoV-2 B.1.1.7 mutant trimeric spike protein with hACE2 as described previously. After confirmation of their trimeric status by SEC-HPLC (Fig. 6C), the sensograms were again fitted using global fitting with the two-state reaction model (Fig. 6A and B).

The dissociation phases of the trimeric spike proteins (Fig. 6A) show biphasic decays as already observed for the monomeric SARS-CoV-2 S1-S2. Again, the secondary state model allowed the most accurate fit for the given sensograms. The component analysis reveals that the secondary state transition (Fig. 6A, blue line) was dominating the complex formation after a short initial contact time. Fig. 6B shows the on-off chart of the primary and secondary interaction mode as fitted for the SARS-CoV (2002), the SARS-CoV-2 wt, and SARS-CoV-2 B1.1.7 trimers. When the *k_a_* and *k_d_* values of the primary interaction are compared with those found for the SARS-CoV-2 S1-S2 and RBD constructs, the 2002 and wt trimeric spikes show values in the same range, implying that the initial binding event is not so much different among these constructs. The B.1.1.7 mutant, however, showed faster association and dissociation for the primary contact. Similarly, the kinetic values of the B.1.1.7 secondary state deviate from those identified for the 2002 and wt trimers. Here, the secondary state transition rate is of highest interest because it will directly impact the contact time that will be needed to form a complex with enhanced stability. The *k_a_* identified for B.1.1.7 is 5.1 and 6.1 faster than the transition rate for the wt and 2002 trimer, respectively.

The significance of this finding becomes obvious when it is transferred to physiological conditions, where the probability of a potential primary contact between the viral spike and the cell surface located hACE2 is limited by the local concentration of the two interaction partners. Hence, a more rapid transition of a low- to high-affinity binding state will increase the complex half-time once a primary contact occurs and therefore increases the infection efficacy as observed for the B.1.1.7 and other mutants.

### Summary and conclusion.

This study helps to understand the basis of the enhanced infectivity that has been observed for SARS-CoV-2 and its derived mutants compared with SARS-CoV (2002). We have demonstrated that a secondary state of the SARS-CoV-2 spike–hACE2 complex exists and that the transition increases the stability of the complex by a factor of ~190 (primary versus secondary state) with an overall *K_D_* of 0.20 nM for the monomeric spike S1-S2 protein (for full list of all kinetic values see Table 1). Furthermore, when the isolated RBD was assayed for its affinity to hACE2, no secondary state formation was observed. This finding clearly suggests that the context of the whole ectodomain is needed to promote the secondary state within the complex after the first contact is mediated by the RBD. When the kinetics of the monomeric SARS-CoV-2 S1-S2 were compared with the trimeric variant, an increased secondary state transition was identified for the trimeric protein, suggesting cooperative effects between the subunits. These cooperative effects are best explained by the dimeric status of hACE-fc (26), which results in the interaction of two RBDs in the “up” position of one trimeric spike. Using the secondary state model, a 5- to 6-fold increased secondary state transition rate was observed for the B.1.1.7 variant compared with that of the wt and 2002 trimeric spike protein. A previous cryo-electron microscopy (cryo-EM) analysis of the spike-hACE2 complex demonstrated that the RBD-hACE2 complex is dynamic relative to the remaining part of the S protein as well as exhibits intrinsic dynamics (27, 28). This structural flexibility leaves room for the here observed secondary state, which is possibly mirrored by the gradually shift of initially more flexible proportions toward a more rigid conformation. Because these conformational changes are not necessarily restricted to the direct binding interface, we refrain from speculations on what residues may be involved.

**TABLE 1:** Kinetic values of spike protein constructs with hACE2^*a*^

Analyte	Expt	Fitting method	k_a1_ [1/M s]	k_d1_ [1/s]	K_D1_ [nM]	k_a2_ [1/M s]	k_d2_ [1/s]	K_D-total_ [nM]	R^2^/Chi^2^ [-]^*b*^
SARS-Cov-2 S1-S2 monomer	BLI (cropped)	1:1 Langmuir	1.1E + 5	2.4E-3	22				0.98
SARS-Cov-2 S1-S2 monomer	BLI (full)	1:1 Langmuir	1.7E + 5	1.9E-3	11				0.96
SARS-Cov-2 S1-S2 monomer	SPR (cropped)	1:1 Langmuir	1.7E + 5	4.7E-3	28.5				0.28 RU^2^
SARS-Cov-2 S1-S2 monomer	SPR (full)	1:1 Langmuir	5.4E + 7	0.7	12.15				6.63 RU^2^
SARS-CoV-2 RBD	SPR	1:1 Langmuir	4.25E + 5	9.1E-3	21.3				0.05 RU^2^
SARS-Cov-2 S1-S2 monomer	SPR	Secondary state	1.8E + 5	6.6E-3	37.5	8.3E-4	8.5E-5	0.2	0.08 RU^2^
SARS-CoV-2 RBD	SPR	1:1 Langmuir	4.3E + 5	9.1E-3	21.3				0.05 RU^2^
SARS-CoV (2002) S1-S2 trimer	SPR	Secondary state	6.7E + 5	4.7E-3	6.7	8.8E-3	2.1E-4	0.16	0.06 RU^2^
SARS-CoV-2 wt S1-S2 trimer	SPR	Secondary state	7.8E + 5	1.4E-3	1.8	1.1E-2	2.4E-4	0.04	0.08 RU^2^
SARS-CoV B.1.1.7 S1-S2 trimer	SPR	Secondary state	1.31 E + 6	2.5 E-2	192.7	5.3E-2	1.1E-4	0.04	0.07 RU^2^

a Measurements were performed using the immobilized hACE2 dimer as the ligand and spike protein constructs as analytes.

b [-] means that RU^2^ and Chi^2^ values have no SI unit.

Taken together, these findings highlight the role of the SARS-CoV-2 spike protein in the context of the ongoing pandemic and stress its importance as a potential drug target. The presented SPR method for the verification of secondary state transitions within the spike protein-hACE2 complex will allow a straightforward way of predicting the infectiousness of newly appearing SARS-CoV-2 variants. To date, several studies aim for the inhibition of the spike-hACE2 interaction by targeting one of the interaction partners (29–32). However, the present study shows that an efficient inhibitor should impact both the primary as well as secondary binding state in order to obtain a significant reduction of complex formation. Since the secondary transition rate is the decisive parameter for a potentially enhanced infection rate, it is important to understand its molecular mechanism. Targeting the secondary state transition may be very efficient for the development of therapeutic compounds for COVID-19. Most importantly, we suggest that future researchers should determine the full quantitative kinetic binding behavior to hACE2 for newly appearing spike protein variants to possibly predict the infectivity efficacy of the underlying virus variant.

## MATERIALS AND METHODS

### Protein expression and purification.

Expression of SARS-Cov-2 spike protein constructs was either performed using High Five insect cells [SARS-CoV-2 RBD, SARS-CoV-2 S1-S2 monomer and trimer, SARS-CoV-2 B.1.1.7 S1-S2 trimer, SARS-CoV (2002) trimer] or HEK293-6E cells (hACE-2) via transient gene expression (33, 34). The High Five (Hi5) insect cell line (officially called BTI-Tn-5B1-4) was isolated by the Boyce Thompson Institute for Plant Research (USA). The cell line was obtained from Thermo Fisher Scientific (USA). The HEK293-6E cell line was licensed from National Research Council (NRC), Biotechnological Research Institute (BRI, Canada). Recombinant protein genes for the CoV constructs (see Fig. S2 in the supplemental material) were synthesized by Genscript (USA) or Thermo Fisher Scientific. Protein samples were purified using HisExcel columns (Cytiva) for 6× His-tagged proteins, StrepTrap HP columns (Cytiva, USA) for TwinStrep-tagged proteins (all trimeric proteins with foldon sequence were Strep- and His-tagged and were purified via StrepTrap HP column), or protein A columns (Thermo Fisher Scientific) for Fc-tagged proteins (see Table 2 for complete list of all constructs and modifications). The C-terminal introduction of a T4 bacteriophage foldon sequence (with exception of the SARS-CoV construct) was used for the induction of S1-S2 stable trimers (35). All purifications steps were performed on Äkta Start or Äkta Pure systems (Cytiva) according to the manufacturer’s protocol. Depending on the protein size, size exclusion chromatography (SEC) was performed as a final polishing step using either a Superdex 200 or Superose 6 column (Cytiva) in 20 mM Tris (pH 8.0) and 150 mM NaCl as the running buffer. For verification of the oligomeric status of the spike protein constructs as well as hACE2, SEC-HPLC was performed. For SEC-HPLC, proteins were diluted to 5 μg/mL and injected on a Bio-SEC3 300 Å column using a 1260 Infinity II system (Agilent, USA) with 1 mL/min phosphate-buffered saline (PBS; pH 7.4) as the running buffer. A full list of all used protein constructs and their origin as well as included modifications are shown in Table 2.

**TABLE 2:** List of SARS-CoV, SARS-CoV-2 spike protein, and hACE2 constructs used in the study

Construct name	Supplier	Expression organism	Tag
SARS-CoV-2 RBD-His	In-house	High Five insect cells	6× His tag
SARS-CoV-2 S1-S2-His monomer	In-house	High Five insect cells	6× His tag
SARS-CoV (2002) S1-S2-His trimer	In-house	High Five insect cells	6× His tag
SARS-CoV-2 S1-S2-His-T4 trimer	In-house	High Five insect cells	6× His tag, T4-foldon
SARS-CoV-2 B.1.1.7 S1-S2 -His-T4 trimer	In-house	High Five insect cells	6× His tag, T4-foldon
hACE2-fc dimer	In-house	HEK293-6E cells	IgG1-Fc
hACE2-His biotin dimer	Acrobiosystems, USA (Cat. No. AC2-H82E6)^*a*^	HEK293 cells	Biotinylated Avitag, 6× His

a UniProt KB Q9BYF1.

### Kinetic experiments biolayer interferometry (BLI).

BLI kinetic experiments were performed with an Octet RED 96 BLI system using streptavidin-coated high-precision SAX-sensors (Sartorius, GE) and a shaking speed of 1,000 rpm. hACE2 was immobilized to a binding level of 1.6 nm with a concentration of 5 μg/mL. Serial dilutions of the S1-S2 SARS-CoV-2 spike protein were prepared in range of 3.9 to 250 nM in 10 mM HEPES (pH 7.4), 150 mM NaCl, 3 mM EDTA, and 0.005% Tween 20. The experiment was performed with double reference subtraction.

### Surface plasmon resonance kinetic experiments.

SPR kinetic experiments were performed with a T200-SPR Biacore system (Cytiva) using a protein A/G-coated sensor chip (PAGD-200M; Xantec) and a flow rate of 30 μL/min unless otherwise noted. hACE2 was captured to a response level of 70 RU for each cycle using a concentration of 2.5 μg/mL. The surface was regenerated with 10 mM NaOH by 2 × 30-s injections at 10 μL/min. For kinetic measurements, serial dilutions of the spike protein were prepared in range from 3.9 to 62.5 nM for the monomeric S1-S2 and 0.65 to 50 nM for the trimeric constructs in 10 mM HEPES (pH 7.4), 150 mM NaCl, 3 mM EDTA, and 0.005% Tween 20. Data fitting was performed using Biacore T200 data evaluation software v. 3.2. For all fits, a contribution of refractive index was excluded.

While testing for the second state interaction, hACE2 was immobilized to a response level of 25 RU for each cycle. A total of 500 nM S1-S2 spike protein was injected with contact times of 150 to 600 s. Buffer referencing was performed prior to each analyte injection cycle. The experimental evaluation was done by alignment of the dissociation start and normalization of the saturation response signal to 100% by the time point of injection phase end.

### Data availability.

The data sets generated during and/or analyzed during the current study are available from the corresponding author on reasonable request.
